# Prognostic value of genomic alterations in head and neck squamous cell carcinoma detected by comparative genomic hybridisation

**DOI:** 10.1038/sj.bjc.6601199

**Published:** 2003-08-26

**Authors:** J N E Ashman, H S Patmore, L T Condon, L Cawkwell, N D Stafford, J Greenman

**Affiliations:** 1 Academic Departments of Otolaryngology and Head and Neck Surgery, University of Hull, Hull HU6 7RX, UK; 2 Academic Department of Oncology, University of Hull, Hull HU6 7RX, UK; 3 Academic Surgical Unit (JG), University of Hull, Hull HU6 7RX, UK

**Keywords:** head and neck squamous cell carcinoma, comparative genomic hybridisation, survival

## Abstract

A total of 45 primary head and neck squamous cell carcinomas were analysed by comparative genomic hybridisation to identify regions of chromosomal deletion and gain. Multiple regions of copy number aberration were identified including gains affecting chromosomes 3q, 8q, 5p, 7q, 12p and 11q and deletion of material from chromosomes 3p, 11q, 4p, 5q, 8p, 10q, 13q and 21. Kaplan–Meier survival analysis revealed significant correlations between gain of 3q25–27 and deletion of 22q with reduced disease-specific survival. In addition, gain of 17q and 20q, deletion of 19p and 22q and amplification of 11q13 were significantly associated with reduced disease-free survival. A Cox proportional hazards regression model identified deletion of 22q as an independent prognostic marker. The data presented here provide further evidence that the creation of a genetically based tumour classification system will soon be possible, complementing current histopathological characterisation.

Head and neck squamous cell carcinoma (HNSCC) is the fifth most common cancer worldwide and contributes significantly to the morbidity and mortality associated with human cancer. Despite numerous advances in treatment the long-term survival rates for HNSCC have changed little over the last 20 years (Jemal *et al*, 2003). Currently, the management of HNSCC is based on the assessment of a variety of clinical and pathological parameters, of which nodal status conveys the strongest prognostic information (O'Brien *et al*, 1986). However, in many instances, these factors fail to predict accurately the clinical behaviour of an individual patient's tumour. A greater understanding of the molecular basis of HNSCC will hopefully allow the biological properties of an individual patient's tumour, including metastatic potential, response to therapy and likelihood of recurrence, to be more precisely determined.

Definition of the genetic changes underlying HNSCC is underway but remains incomplete. Classical cytogenetic analysis of HNSCC tumours has revealed multiple chromosomal abnormalities and highly complex karyotypes. The most widely reported aberration demonstrating a correlation with clinical outcome is amplification of chromosome 11q13. Both the amplification of this band and overexpression of the *cyclin D1* oncogene have been repeatedly associated with poor prognosis (Akervall *et al*, 1995; Meredith *et al*, 1995). Many of the limitations of conventional cytogenetic analysis have been overcome with the development of molecular techniques including FISH and comparative genomic hybridisation (CGH). Comparative genomic hybridisation is a powerful technique that screens the entire tumour genome for DNA sequence copy number alterations. Comparative genomic hybridisation has previously been applied in several HNSCC studies and has demonstrated a nonrandom pattern of genomic aberrations including deletions of material from 3p, 4q, 5q, 9p, 18q and gains involving 3q, 5p, 7p, 8q, 11q, 17q and 20q (reviewed in Struski *et al*, 2002).

In this study, we have applied CGH to HNSCC patients in order to characterise the chromosomal imbalances underlying this disease and evaluated the prognostic significance of the aberrations identified.

## METHODS

### Patients

Informed consent was obtained from all patients and local ethical approval granted for the study. In total, 53 consecutive patients undergoing surgery for a single primary HNSCC between April 1996 and November 2001 were included in this study. Biopsy-sized specimens were obtained at the time of surgical resection, snap frozen in theatre and stored at −80°C until analysis. Specimens were taken immediately adjacent to specimen sent for histopathological assessment and all samples were analysed by a single pathologist. Several patients were excluded from the study cohort for the following reasons: only specimens containing >70% malignant cells upon pathological examination were included, as CGH analysis requires that the tumour cells constitute the majority of the tissue specimen (*n*=5 excluded). Patients who received radiotherapy prior to surgical resection were excluded from the cohort (*n*=3 excluded) to avoid the inclusion of specimens containing radiotherapy-induced genetic changes. None of the 53 patients had received preoperative chemotherapy. The clinicopathological details of the 45 patients analysed are detailed in Table 1

**Table 1:** Clinicopathological details of the 45 HNSCC patients included in this study

	**Total**	**Alive**	**Dead of disease**
*No. of patients*	45	28	17
Male	35	22	13
Female	10	6	4

*Tumour site*			
Larynx	21	15	6
Hypopharynx	13	9	4
Oropharynx/oral cavity	10	4	6
Parotid	1	0	1

*Stage*			
T1	1	1	0
T2	12	8	4
T3	9	8	1
T4	23	11	12
N0	15	13	2
N+	30	15	15
N1	8	5	3
N2	20	10	10
N3	2	0	2

*Differentiation*			
Well	3	2	1
Moderate	18	11	7
Poor	22	14	8
Undifferentiated	2	1	1

N0=no nodal involvement; N+=with nodal involvement.

.

### Comparative genomic hybridisation

DNA was extracted from either serial 20 *μ*m cryostat sections or whole biopsy by proteinase K digestion followed by phenol–chloroform extraction. DNA quality and purity were assessed by electrophoresis and spectrophotometry at 260 nm, and CGH was performed essentially as described previously (Stafford *et al*, 1999). All CGH reagents were obtained from Abbott Laboratories Ltd (Maidenhead, UK). All experiments were performed in combination with both positive (DNA with known aberrations) and negative (normal : normal hybridisation) control experiments. Sex mismatching of test and reference DNA precluded the analysis of the sex chromosomes. Deletions and gains of DNA were identified whenever the CGH ratio profile exceeded thresholds established through normal : normal hybridisations (0.85 and 1.15 respectively).

### Statistical analysis

Disease-specific survival time was calculated as the time between the date of tissue acquisition (surgical resection) and death. Only patients with cancer-specific death were included in the analysis and patients alive at the time of last follow-up were censored accordingly. Disease-free survival was defined as the time between tissue acquisition and evidence of disease recurrence. For purposes of statistical analysis local, regional or distant recurrence were grouped together as disease recurrence. Survival analysis was performed using the Kaplan–Meier method and the difference in survival curves was tested for statistical significance using the log rank test. Cox proportional hazard analysis was used to test variables identified by Kaplan–Meier analysis for independent prognostic significance. The χ^2^ test was used to evaluate relationships between categories. All statistical analysis was performed using the SPSS version 10 software package and values of *P*<0.05 were considered to be statistically significant.

## RESULTS

### Overview of genomic changes in HNSCC

**Figure 1:**
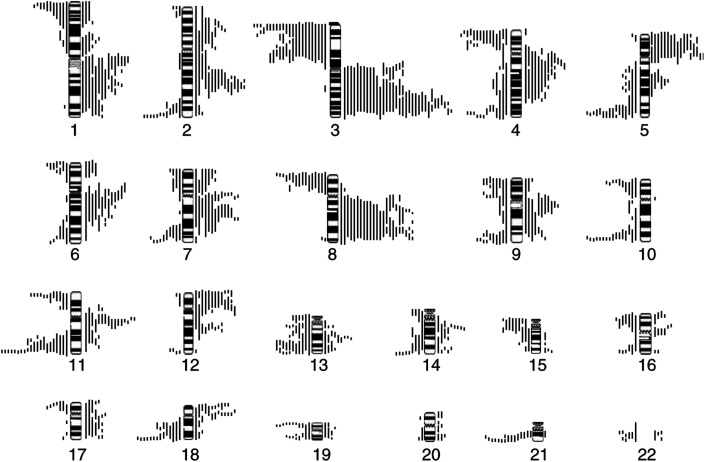
Summary of all chromosomal gains and losses identified in 45 primary HNSCC tumours by CGH. Lines to the left of the chromosome ideograms represent regions of deletion and lines to the right represent gains. The relative lengths of each line represents the size of the region of gain or loss. All CGH ratio deviations identified using the thresholds detailed in the methods were included. A few, particularly small, regions of copy number change below the accepted resolution of CGH (∼10 Mb) are present and care should be taken in interpreting these.

Genetic aberrations were detected in all 45 specimens analysed and occurred across the entire genome. Figure 1

summarises all the chromosomal aberrations identified. Overall, CGH identified a complex pattern of aberrations; however, several frequent chromosomal loci of copy number change were seen. These included gains affecting chromosomes 3q, 8q, 5p, 7q, 12p and 11q and deletion of material from chromosomes 3p, 11q, 4p, 5q, 8p, 10q, 13q and 21. Those occurring in >20% of the cohort of tumours analysed are highlighted in Table 2

**Table 2:** Frequencies of copy number imbalances identified in this study (>20% of cases), ranked according to the most frequently occurring

**Chromosome**	**Aberration**	**Frequency (%)**
3q25–q27	Gain	75.5
3q21–q24	Gain	62.2
8q22.2–qter	Gain	55.6
3p24–pter	Deletion	51.1
11q23.1–qter	Deletion	48.9
8q21.1–q22.2	Gain	48.9
3qcen–q13.3	Gain	46.7
8qcen–q21.1	Gain	46.7
5p	Gain	44.4
4p14.1–pter	Deletion	42.2
3p13–p13.3	Deletion	40.0
5q32–qter	Deletion	37.8
8p12–pter	Deletion	35.6
7p12–q11.23	Gain	35.6
12p	Gain	35.6
11q13.1–q14.1	Gain	35.5
21	Deletion	35.5
3p21.3–p23	Deletion	33.3
10q24.1–qter	Deletion	33.3
13q22–qter	Deletion	33.3
1q23–q31	Gain	31.1
4qcen–q21.3	Gain	31.1
4q32–qter	Deletion	28.9
18q22–qter	Deletion	28.9
9q33–qter	Deletion	26.7
6qcen–q16.1	Gain	24.4
1p35–pter	Deletion	24.4
11p14–pter	Deletion	24.4
2q36–qter	Deletion	24.4
18q11.2	Gain	24.4
9q13–q22.2	Gain	22.2
18p	Gain	22.2
19p13.2–pter	Deletion	22.2
7q31.3–qter	Deletion	22.2
10p14–qter	Deletion	22.2
12q21.2–q21.3	Gain	22.2
14q13–q21	Gain	20.0
9p21–pter	Deletion	20.0
15q11.2–q15	Deletion	20.0

and are consistent with previously published CGH analyses of HNSCC. There was a mean of 18.8 aberrations per tumour (range 4–38), 9.4 deletions (range 1–23) and 9.5 gains (range 1–18). Amplifications (CGH ratio >1.5) were detected on 3q (10 cases), 11q (four cases), 5p (two cases), 7p and 8q (one case each). There was no significant difference between the total number of copy number aberrations when patients were stratified by T stage, nodal involvement, tumour stage or differentiation. Only gain of 7p and deletion of 9q were significantly more frequent in patients with nodal involvement (*P*=0.019 and 0.012 respectively) and gain of 20q significantly associated with recurrent disease (*P*=0.022).

### Clinical outcome

**Figure 2:**
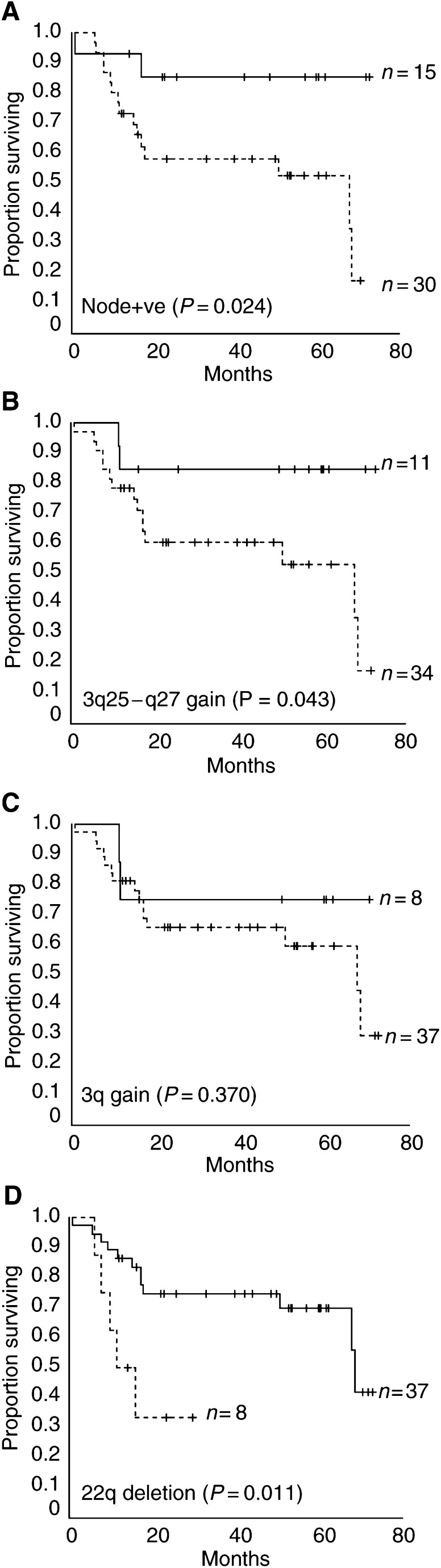
Kaplan–Meier survival curves for disease-specific survival in tumours demonstrating (**A**) tumours with pathologically proven nodal involvement at time of surgery, (**B**) gains specifically within the region 3q25–q27, (**C**) gains on 3q and (**D**) deletion of 22q. Dotted lines represent tumours that exhibited the chromosomal imbalance (or nodal involvement in the case of **A**) and solid lines represent those that did not. Crosses indicate censored cases (patients alive at time of analysis).

Follow-up of the patients was continued until November 2002 and was available for all patients. The mean duration of follow-up was 35.0 months overall (95% confidence interval (CI), 28.3–41.7). During this period, 37.8% of patients died from their disease (mean time to death 20.0 months, 95% CI, 10.0–29.9) and 51.1% patients developed disease recurrence (including six local, 14 regional, one local and regional and two distant recurrences; mean time to disease recurrence 14.2 months, 95% CI, 8.6–19.7). Mean follow-up for patients remaining alive at time of analysis was 44.0 months (95% CI, 36.9–51.2). The clinical outcome for patients with pathologically proven regional lymph node metastases was significantly worse than for patients with no nodal involvement with a mean survival of 43.9 months (95% CI, 33.5–54.2) and 63.9 months (95% CI, 51.1–75.4) respectively (*P*=0.024; Figure 2A

). When this analysis was extended to include patients who were not initially submitted for a neck dissection, but eventually presented with a neck recurrence (*n*=3 additional tumours), this association was slightly stronger with a mean survival of 44.4 months (95% CI, 34.6–54.1) for all patients with nodal involvement and 66.7 months (95% CI, 55.5–78.0) for patients in which the regional lymph nodes remained disease-free through the course of follow-up (*P*=0.021). The clinical and follow-up details of the eight patients excluded for reasons detailed in the Methods section did not differ significantly from the cohort of 45 patients for which genetic analysis was performed (data not shown).

### Correlation of CGH data with survival

When disease-free survival was analysed, several aberrations demonstrated significant associations (Table 3

**Table 3:** Chromosomal aberrations demonstrating a significant association with disease-free (upper) and disease-specific survival (lower)

				**Mean survival (months)**	
**Chromosome**	**Aberration**	**Log rank (*P*)**	**No. of cases with aberration**	**With aberration**	**Without aberration**
*Disease-free survival*					
Nodal status	—	0.039	30	(N+) 33.4	(N0) 54.2
17q	Gain	0.049	9	10.9	44.4
20q	Gain	0.001	5	4.6	46.1
19p	Deletion	0.002	10	13.5	44.9
22q	Deletion	0.004	8	8.7	45.2
11q13	Amplification	0.001	4	3.7	44.1

*Disease-specific survival*					
Nodal status (at surgery)	—	0.024	30	(N+) 43.9	(N0) 63.9
3q25–27	Gain	0.043	34	44.9	63.3
22q	Deletion	0.011	8	17.0	54.9

N+=with nodal involvement; N0=without nodal involvement.

). Gain of 3q25–27 and losses on 22q were the only aberrations significantly associated with disease-specific survival (Table 3, Figure 2). The mean survival for patients with gain of the chromosomal locus 3q25–q27 was significantly reduced (44.9 months) when compared to those without (63.3 months) (*P*=0.04; Figure 2B). Interestingly, this significance did not hold when patients with gains anywhere on 3q were compared with those without (*P*=0.37; Figure 2C). The association of deletion of 22q with reduced survival was stronger (*P*=0.01; Figure 2D); however, only eight patients in the cohort demonstrated this deletion. Mean survival for patients with loss of 22q was 17.0 months compared with 54.9 months for those without. Cox proportional hazard analysis indicated that nodal status and deletion on 22q were independent prognostic variables (Table 4

**Table 4:** Cox proportional hazard analysis

**Variable**	**Cox hazard ratio (95% CL)**	***P*-value**
Nodal status	4.42 (1.00–19.44)	0.049
22q deletion	3.19 (1.04–9.80)	0.042
3q25–27 gain	3.27 (0.73–14.58)	0.121

).

## DISCUSSION

Comparative genomic hybridisation analysis of this cohort of tumours has provided further evidence for the importance of gain of material on 3q in the development of HNSCC. The gain of 3q25–27 was the most prevalent gain detected in this cohort of primary HNSCC. Kaplan–Meier survival analysis demonstrated a significant association of 3q25–27 gain with reduced overall survival; however, Cox regression analysis indicated this effect was not independent. Several other CGH studies have identified 3q as a consistent site of both gain of DNA and amplification (Bergamo *et al*, 2000; Bockmühl *et al*, 2000; Hashimoto *et al*, 2001; Hermsen *et al*, 2001; Singh *et al*, 2001). A similar association with survival has previously been identified by Bockmühl *et al* (2000). In their study of 113 primary HNSCC tumours, gain of material at 3q21–q29 demonstrated a strong association (*P*=0.006–log rank test) with a reduced overall survival. The significance of the difference between the region of chromosome 3q identified in this cohort (3q25–27) and Bockmühl study (3q21–29) is unclear. Both studies involved heterogenous populations of HNSCC tumours, a major limitation of any HNSCC analysis; however, the overall sub-site distribution was similar. The observed difference may simply reflect the different methods of analysis employed. Bockmühl *et al* used a CGH analysis software package that allowed integrated statistical analysis of chromosomal alterations at each chromosomal band throughout the genome (at the ISCN 400 band resolution). In the cohort presented here, the region 3q25–27 represents the minimal region of overlap present in all tumours demonstrating a gain on 3q2.

The gain of 3q has been reported in numerous tumour types, most frequently in those of squamous origin, including HNSCC, squamous lung carcinomas and cervical carcinoma. (Chujo *et al*, 2002; Struski, 2002). In squamous cell carcinoma of the uterine cervix, the gain of 3q has been shown to define the transition from severe dysplasia to invasive carcinoma (Heselmeyer *et al*, 1996). The high frequency of 3q gains identified in the present study is consistent with 3q gain being an early event in HNSCC tumorigenesis. However, an early, essential, genetic requirement for tumorigenesis would be unlikely to carry prognostic value as demonstrated here and previously (Bockmühl *et al*, 2000). Recently, Hashimoto *et al* (2001) have further delineated the involvement of 3q gains in HNSCC tumorigenesis by demonstrating a correlation between amplification, as opposed to low level gain, of 3q26–qter and tumour progression. Hashimoto identified gain of 3q26–qter in 91% of tumours (*n*=32), and amplification at a significantly higher frequency in T4 tumours (70%) when compared with T2 and T3 tumours (both 27%; *P*<0.05–Fisher's exact test). Using interphase FISH, Singh *et al* (2002) demonstrated a significant increase in 3q26–q27 amplification from normal mucosa (3%), premalignant mucosa (25%) to invasive cancer (56%; *P*<0.01). This study also showed a significant increase in tumour recurrence and cancer-related death when tumours were stratified by 3q26–q27 copy number (normal, low-level and high-level (>4 ×) amplification). It appears likely that multiple genes within the region of 3q gain identified in this study are important for HNSCC progression. Alterations in a gene(s) early in HNSCC tumorigenesis may occur through low-level gains, while amplification of additional genes may occur later in the progression of HNSCC and perhaps characterise more aggressive tumours with a worse prognosis.

Several candidate genes have been localised to 3q25–q27 including the *FGF12* growth factor, *cyclin L* and *PIK3CA* (Redon *et al*, 2001, 2002). *PIK3CA* encodes the p110 catalytic subunit of phosphatidylinositol-3′-kinase, a critical component of many cell signalling pathways including those of *EGF* and *PDGF* (Volinia *et al*, 1994). Targeted FISH experiments using yeast artificial chromosomes (YACs) have demonstrated amplification of the *PIK3CA* gene in a panel of HNSCC cell lines (Singh *et al*, 2001). Increased copy number of the *PIK3CA* gene has also been identified in cervical cancer and ovarian cancer, and in the latter case has been shown to be associated with increased expression (Shayesteh *et al*, 1999; Ma *et al*, 2000).

The literature on the involvement of 22q in HNSCC progression is limited. Comparative genomic hybridisation analysis has demonstrated copy number changes in up to 50% of cases analysed with both deletion and, more commonly, gain of material from 22q having been reported. Preliminary LOH data have proposed that allelic imbalance on 22q is more frequent in laryngeal and oral tumours than other HNSCC (dos Reis *et al*, 2002). Comparative genomic hybridisation analysis of 22q, along with regions of 1p, 9p, 16 and 19 can be problematic and should be interpreted with caution (Jeuken *et al*, 2002). Particularly stringent evaluation of simultaneous negative control experiments is required to identify experimental artefact within this region. All relevant negative control experiments were of a high quality and did not demonstrate false-positive aberrations. In the 45 HNSCC tumours reported here, deletion of 22q was a relatively infrequent finding occurring in only eight cases; however, the clinical outcome for these patients was particularly poor. Recently, a study of 40 primary oral tumours by quantitative reverse transcriptase–polymerase chain reaction (RT–PCR) identified deletion of the *DIA 1* gene, a cytochrome *b*5 reductase, at 22q13 in 25% of cases and this loss was also significantly associated with a decrease in survival (*P*=0.0018–log rank test; Reis *et al*, 2002).

One surprising result from this analysis was the low frequency of 11q13 amplification detected in this cohort of tumours (four out of 45), compared with other studies. To some extent, this may reflect potential dilution of the CGH signal by normal cell contamination as well as the arbitrary discrimination between gain (CGH ratio 1.15–1.5) and amplification (ratio>1.5). Unlike Bockmühl *et al* (2000), no association between gain of 11q13 and reduced survival was identified; however, when tumours demonstrating amplification of this region were analysed separately a strong association with reduced disease-free survival was revealed. This association with 11q13 amplification and poor prognosis is consistent with previous classical karyotypic data (Akervall *et al*, 1995; Meredith *et al*, 1995).

The demonstration of genetic aberrations exhibiting prognostic significance in this relatively small cohort of tumours, using a low-resolution technology, provides strong encouragement for the continued investigation of the molecular abnormalities underlying HNSCC and other tumours. Such findings demonstrate that molecular characterisation of HNSCC can provide additional markers of prognosis to supplement the classical pathological assessment of tumours. It is important to emphasise that, as with many other HNSCC studies, this cohort consisted of a mixed population of tumour subsites. This fact does not detract from the significance of the findings presented here, but suggests that additional studies on homogeneous populations of HNSCC tumours may reveal additional subsite-specific genetic markers of prognosis, which are masked when analysed as a single entity.

Many of the chromosomal regions identified in this study contain tumour genes already implicated in the tumorigenesis of HNSCC including 3p14 (*fhit*), 7p12 (*EGFR*), 7q31 (*ING3*), 8q24 (c*-myc*), 9p (*p16*), 11q13 (*ccnd1*) and 17p (*p53*). The affected genes in other regions identified in this, and other studies, remain to be fully elucidated. The body of knowledge of genetic aberrations in HNSCC is rapidly growing, and with the advent of DNA microarray technology the copy number and gene expression levels of hundreds of genes can be accurately established. Performing CGH on an ordered array of genomic clones, in place of metaphase chromosomes, dramatically increases the resolution of the technique. This modification of the CGH method has demonstrated copy number changes as low as 100 kb (Solinas-Toldo *et al*, 1997). Such a significant increase in resolution and sensitivity may help resolve discrepancies between CGH studies, that is, regions such as 11q13 that correlate with prognosis in some studies but not others. Microarray-based strategies represent the future of gene copy number analysis in HNSCC and the identification of chromosomal regions with prognostic importance will facilitate the design of such higher resolution strategies, allowing further molecular characterisation of this disease.
